# High Bacterial Agglutination Activity in a Single-CRD C-Type Lectin from *Spodoptera exigua* (Lepidoptera: Noctuidae)

**DOI:** 10.3390/bios7010012

**Published:** 2017-03-01

**Authors:** Leila Gasmi, Juan Ferré, Salvador Herrero

**Affiliations:** ERI de Biotecnología y Biomedicina (BIOTECMED), Department of Genetics, Universitat de València, 46100 Burjassot, Spain; gasmi.leila@gmail.com (L.G.); ferrej@uv.es (J.F.)

**Keywords:** C-type lectin, agglutination, CRD, bacterial detection, *E. coli*

## Abstract

Lectins are carbohydrate-interacting proteins that play a pivotal role in multiple physiological and developmental aspects of all organisms. They can specifically interact with different bacterial and viral pathogens through carbohydrate-recognition domains (CRD). In addition, lectins are also of biotechnological interest because of their potential use as biosensors for capturing and identifying bacterial species. In this work, three C-type lectins from the Lepidoptera *Spodoptera exigua* were produced as recombinant proteins and their bacterial agglutination properties were characterized. The lowest protein concentration producing bacterial agglutination against a panel of different Gram+ and Gram− as well as their carbohydrate binding specificities was determined for the three lectins. One of these lectins, BLL2, was able to agglutinate cells from a broad range of bacterial species at an extremely low concentration, becoming a very interesting protein to be used as a biosensor or for other biotechnological applications involving bacterial capture.

## 1. Introduction

Lectins are carbohydrate-interacting proteins that play a pivotal role in multiple physiological and developmental aspects of all organisms. In the case of the immune defense, lectins are mainly involved in two important processes: the recognition of the pathogen, and the cellular interactions that lead to pathogen neutralization [1]. Based on their functions, their structural characteristics, and their carbohydrate specificity, lectins can be grouped into several families [2]. One of the largest families is the one formed by the Ca^2+^-dependent lectins, also known as C-type lectins. This family is defined by the presence of a structural motif, the C-type lectin domain (CTLD), regardless of their ability to bind sugars [3].

Interaction of the lectins with the different bacterial and viral pathogens is mediated by the presence in their structure of carbohydrate-recognition domains (CRD). Each CRD forms a double-loop structure stabilized by two highly conserved disulfide bridges, located at the base of the loops [4], which mediate the specific binding to sugars [1]. In addition, these proteins often act in an oligomeric form, which increases their avidity for multivalent ligands [3]. Lectin-bacterial interaction is mediated by binding to certain carbohydrates in the cell surface and is easily recognized by the bacterial clumping (agglutination) that occurs when the lectin is added to the bacterial suspension. In the case of C-type lectin, the bacterial agglutination also requires the presence of Calcium ions (Ca^2+^) [2].

In the case of the invertebrates, due to the lack of an acquired immune system, their defense against pathogens relies mainly on their innate immunity [5]. One of the basic actions in the innate immunity is the production of a large set of lectins that are involved in multiple processes such as phagocytosis, encapsulation, nodule formation, hemolymph coagulation, and prophenoloxidase cascade [6,7,8]. An additional function of C-type lectins in invertebrates has been reported in parasitoid wasps. During the parasitic process, the wasp injects its eggs into the insect host bodies or eggs. The parasitoid egg develops inside their host. The survival and appropriate development of the parasite also depends on the co-injection, together with the eggs, of polydnaviruses (viral particle or viral products) that block the immune defence of the host and prevent the killing of the parasite [9]. Among the viral-derived products that contribute to the wasp parasitism, several viral C-type lectins participate in the process by binding to the parasitoid eggs, suggesting a likely role of these lectins in hiding the egg from the host immune system [10,11].

In our previous studies, we have identified a large set of C-type lectins in the transcriptome of the lepidopteran *Spodoptera exigua* [12]. In contrast to most of the Lepidoptera C-type lectins, which contain two CRD in their structure, some of these lectins, named bracovirus-like lectins (BLLs), have a single CRD and are proposed to be derived by horizontal gene transfer events from bracoviruses (a type of polydnaviruses) and further domesticated by the host [13].

One of these bracovirus-derived C-type lectins (BLL2) has been recently studied and its role in the insect’s immunity has been suggested [13]. During the course of that study, we observed that BLL2 was able to induce *Escherichia coli* agglutination at a relative low concentration. Based on this observation, we decided to explore in more detail its agglutination properties and see whether such a property was restricted to this particular lectin or could be extended to other BLLs. In this work, we have characterized the agglutination properties of BLL2 and two additional BLL members against a broad range of bacteria, confirming the unique properties of this C-type lectin.

## 2. Materials and Methods

### 2.1. Bacterial Strains

Gram-negative (Gram−) and Gram-positive (Gram+) bacteria expressing fluorescent proteins were initially used to perform the agglutination tests. *Escherichia coli* Top10 (Invitrogen) expressing the red fluorescent protein dt-tomato was kindly provided by Rafael Vázquez-Manrique (Hospital La Fe, Valencia, Spain). *Bacillus thuringiensis* expressing the green fluorescent protein GFP was kindly supplied by Colin Berry (Cardiff University, UK). Additional bacteria used in this study were obtained from the Spanish Type Culture Collection (CECT). As representatives of Gram− bacteria, *E. coli* serotype O157:H7 (CECT 4972), *Pseudomonas aeruginosa* (CECT 110), and *Salmonella enterica* subsp. enterica serovar Abony (CECT 545) were used. As Gram+ bacteria, *Bacillus cereus* (CECT 148), *Listeria monocytogenes* (CECT 4031), and *Staphylococcus aureus* subsp. aureus (CECT 86) were used.

### 2.2. Cloning and Recombinant Expression of Lectins

Specific primers were designed to amplify the complete open reading frames (ORFs) of *Se-BLL1*, *Se-BLL2*, and *Se-BLL3* from cDNA obtained from the fat body of a pool of five fifth instar *S. exigua* larvae (FRA colony). Those primers were designed to add an *Xba*I restriction site at the 5′ end of the gene and an *Xho*I site at the 3′ side. A sequence for 6×His-tag was also included in the reverse primer (C-terminus of the expressed protein). After digestions of the PCR products with *Xba*I and *Xho*I, the DNAs were ligated into the expression vector pET-16b and transformed into *E. coli* XL-Blue competent cells. Selected clones were confirmed by standard Sanger sequencing (NCBI accession numbers: BLL1_FRA, KY111296; BLL2_FRA, KY111297; BLL3, KP406771). After selection of positive recombinant clones, pET-16b_BLL1, pET-16b_BLL2, and pET-16b_BLL3 were transformed into *E. coli* BL21 (DE3) pLysS competent cells. For the production of recombinant proteins, the overnight cultures (15 mL) from an individual colony were inoculated to 1.5 L of LB medium and incubated at 37 °C in an orbital shaker until the OD_600_ reached 0.8–1. Then, isopropyl β-d-1-thiogalactopyranoside (IPTG) was added to a final concentration of 1 mM and incubated overnight with agitation.

To confirm the expression of lectins, bacterial cells were harvested by centrifugation (15 min, 12,000× *g*, 4 °C). Portions of the pellets were resuspended in lysis buffer (20 mM phosphate buffer, pH 7.4, 500 mM NaCl, 3 mg/mL lysozyme, 10 µg/mL DNAse, and 100 µM phenylmethylsulfonyl fluoride). After thirty minutes of incubation, the lysates were centrifuged as above. Supernatants and pellets were separated, total proteins in both fractions were measured by the Bradford method, and the lectins’ expression was confirmed by Western blot using antibodies against the 6 × His tag (BD Pharmingen, San Diego, CA, USA).

### 2.3. Purification of Lectins

Lectins were expressed in insoluble form and thus, solubilisation was required before their purification by affinity chromatography. For solubilisation of the lectins, bacterial cells that had been harvested by centrifugation (15 min, 12,000× *g*, 4 °C) were then resuspended in 20 mM Tris-HCl (pH 8) and lysed by sonication for one minute on ice. After centrifugation in the same conditions, the supernatant was kept and the pellet, containing the insoluble form, was frozen overnight at −80 °C. The next day, the pellet was resuspended in 20 mM Tris-HCl (pH 8) and subjected to sonication as before. The supernatant was saved and the pellet was resuspended again in the same buffer and sonicated again for one minute. This procedure was repeated ten times (modified from [14]). Finally, the supernatants were individually subjected to Western blot. The supernatants with the highest concentration of lectin were selected for purification using the HiTrap™ Chelating HP column (GE Healthcare) operated with a peristaltic pump. The selected supernatants were pooled and then diluted 1:5 (v/v) in the equilibration buffer (20 mM Tris-HCl, pH 8, containing 0.5 M NaCl, 20 mM imidazole, 0.5 mM β-mercaptoethanol, and 6 M urea) and loaded onto the column. Proteins retained in the column were refolded with a decreasing urea stepwise gradient (6 M to 0 M urea in 20 mM Tris-HCl, 20 mM imidazole, 0.5 M NaCl, and 0.5 mM β-mercaptoethanol, pH 8). The retained proteins were eluted with an imidazole stepwise gradient (50 mM to 500 mM imidazole in 20 mM Tris-HCl, 0.5 M NaCl, 0.5 mM β-mercaptoethanol). Aliquots of the elution fractions were analysed in 12% SDS-PAGE and detected by staining with Coomassie blue. The fractions containing the purified lectins were pooled, dialyzed overnight against 20 mM Tris-HCl (pH 8), quantified by the Bradford method, and stored at −20 °C for further experiments.

### 2.4. Agglutination Assay

To assess the agglutination activity of the different lectins, bacteria were collected at mid-logarithmic phase by centrifugation at 6000 rpm for 5 min and the obtained pellets were resuspended in Tris buffer (20 mM Tris-HCl, pH 8.0) at a concentration of about 10^9^ cells/mL. *Escherichia coli* and *B. thuringiensis* expressing the fluorescent proteins were directly used in the agglutination assay. However, the other six bacteria were previously stained by incubation with acridine orange (Sigma; 30 µg/mL) for 20 min at room temperature and washed three times in Tris buffer before being used in this assay.

For the agglutination assay, BLL1, BLL2, and BLL3 were serially diluted (1/10 factor) in Tris buffer and 45 µL aliquots of each lectin were mixed with 45 µL of the bacteria suspension. After incubation for 1 h at room temperature in the presence or the absence of 10 mM CaCl_2_, samples were observed and photographed under a fluorescent microscope (DMI3000, Leica Microsystems, Wetzlar, Germany).

### 2.5. Sugar Binding Specificity

The carbohydrate binding specificities of the three lectins were determined by the agglutination inhibition assay. Nine sugars were tested: D(+)-trehalose dehydrate, D(+)-maltose monohydrate, α-lactose, α-mannose, D(+)-galactose, D(+)-galactose and *N*-acetyl-d-galactosamine (GalNAc), sucrose, and fructose. BLL1, BLL2, and BLL3 were incubated, at a concentration able to agglutinate either *E. coli-*dt-tomato or *B. thuringiensis*-GFP, for 1 h at room temperature with different concentrations of each sugar. Then, the bacterial suspensions were added (in 10 mM CaCl_2_) to test whether the agglutination was inhibited. The inhibitory capacity of the sugar was defined as the minimal concentration able to inhibit the agglutination.

## 3. Results

**Figure 1:**
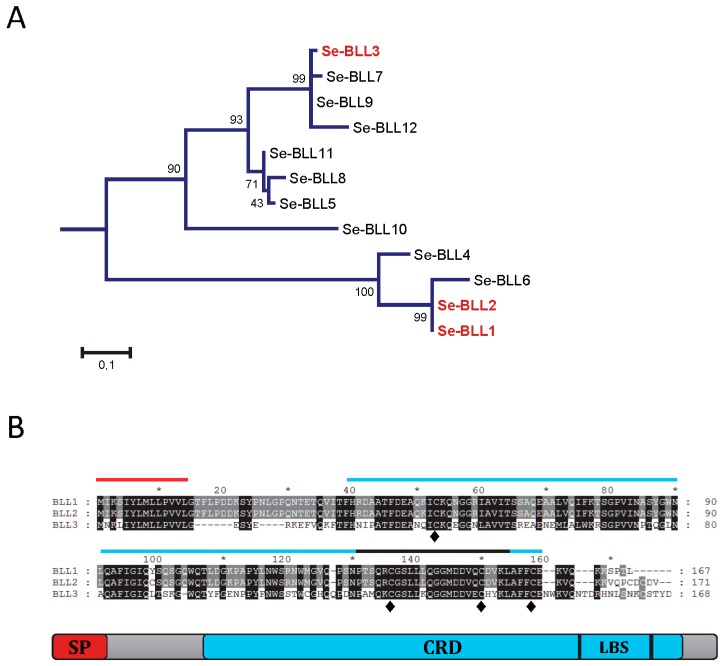
Amino acidic sequences of the bracovirus-like lectins (BLL). (**A**) Phylogenetic relationship of the different BLLs detected in the transcriptome of *Spodoptera exigua*. Phylogenetic distance was calculated by the Maximum-likelihood method. Reliability of an inferred tree was determined using the bootstrap test (1000 replicates). For a clearer view of the branches, bootstrap values are reported over 100; (**B**) Amino acidic alignment of the three BLLs studied, indicating the presence of the predicted signal peptide (SP), the carbohydrate recognition domain (CRD), the ligand binding surface (LBS), and the conserved cysteine residues (♦).

Three different BLLs from *S. exigua* were selected to analyze their ability to interact with bacterial pathogens. From a phylogenetic point of view, two of them (BLL1 and BLL2) are closely related and the third one (BLL3) is more distantly related (Figure 1A). The amino acid sequence for BLL1 and BLL2 was very similar (96% of amino acid identity), while that of BLL3 was less related (54% of amino acid identity). The three proteins had a similar size and architecture: An N-terminal signal peptide, a single carbohydrate binding domain (CBD), and the ligand binding surface (LBS) at the C-terminus (Figure 1B). The three lectins also contained four conserved cysteine residues at the CRD, possibly involved in the stabilization of the CRD by forming two disulfide bridges and hypothetically also involved in protein oligomerization. Interestingly, one of the few residues that differ between BLL1 and BLL2 is a cysteine residue in the CRD of BLL2.

**Figure 2:**
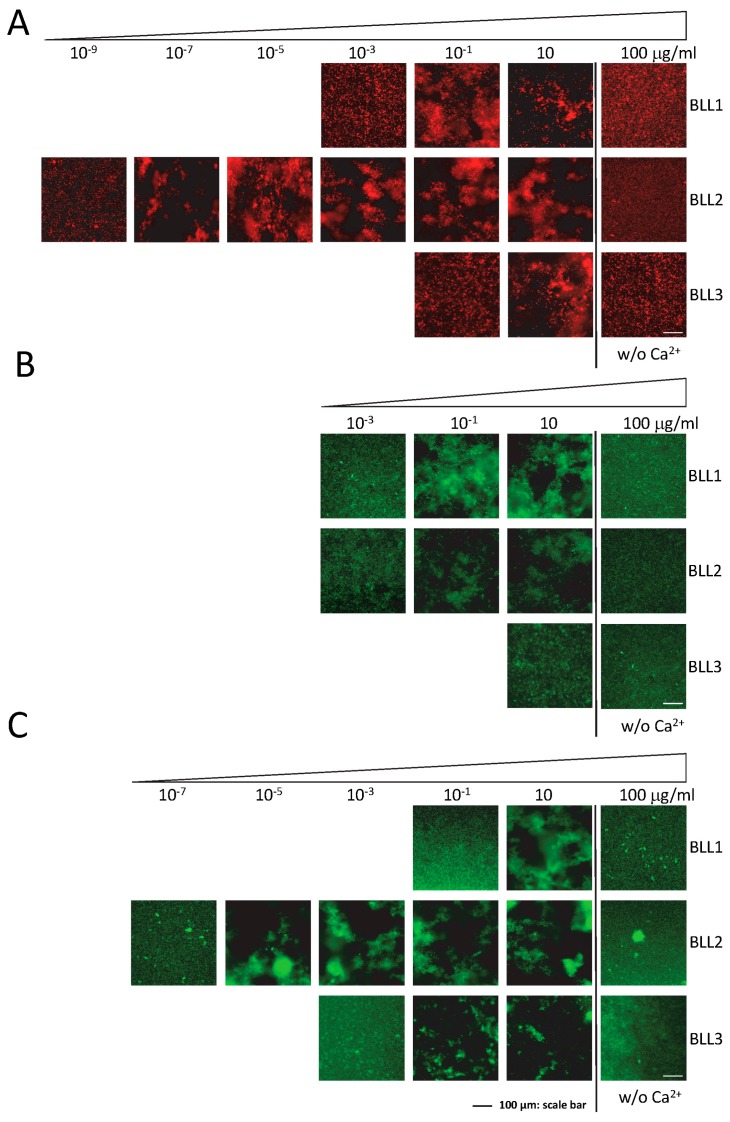
Bacterial agglutination in the presence of the BLLs. Representative images of the agglutination of three types of bacteria ((**A**) *E. coli dt-tomato*; (**B**) *B. thuringiensis GFP*; (**C**) *P. aeruginosa*) in the presence of serial dilutions of the three BLLs. Controls of agglutination in the absence of Ca^2+^ (w/o Ca^2+^) are also shown.

One of the main characteristics of lectins is their ability to recognize pathogens by interacting with their cellular surface. The agglutination assay was performed to evaluate the specific interactions of the lectins in a panel of different Gram+ and Gram− bacterial species (Figure 2). Although with different sensitivities and specificities, the three lectins were able to produce bacterial agglutination in our experimental conditions (Table 1). In concordance with the nature of the three lectins tested (C-type), agglutination required the presence of Ca^2+^ in the medium (Figure 2). The BLL2 showed the highest sensitivity producing agglutination of an *E. coli* strain at a concentration as low as 1 × 10^−8^ µg/mL (0.5 fM) (Figure 2A and Table 1). The BLL2 capacity of producing agglutination on *E. coli* was confirmed with a different strain of *E. coli* and using a different method for the fluorescent detection of the agglutinated bacteria. In this case, the minimum concentration for agglutination was 1 × 10^−7^ µg/mL. BLL2 was also able to agglutinate, at very low concentrations, with other Gram− bacteria such as *P. aeruginosa* or *S. enterica* (Figure 2B and Table 1). Although BLL2 was also able to produce agglutination with Gram+ bacteria, the concentrations needed were in general higher than for the Gram− bacteria, except for *B. cereus* (1 × 10^−4^ µg/mL) (Figure 2B and Table 1).

In contrast to the exceptionally high activity of BLL2 for bacterial agglutination, BLL1 and BLL3 showed agglutinating activity at more standard concentrations (0.01–1 µg/mL) [15,16,17,18]. Between the two of them, BLL1 showed the lowest values of minimal concentration for the Gram− and Gram+ species tested. The BLL3 was in general only able to produce agglutination of Gram+ bacteria at concentrations much higher than those needed to agglutinate Gram− bacteria.

**Figure 3:**
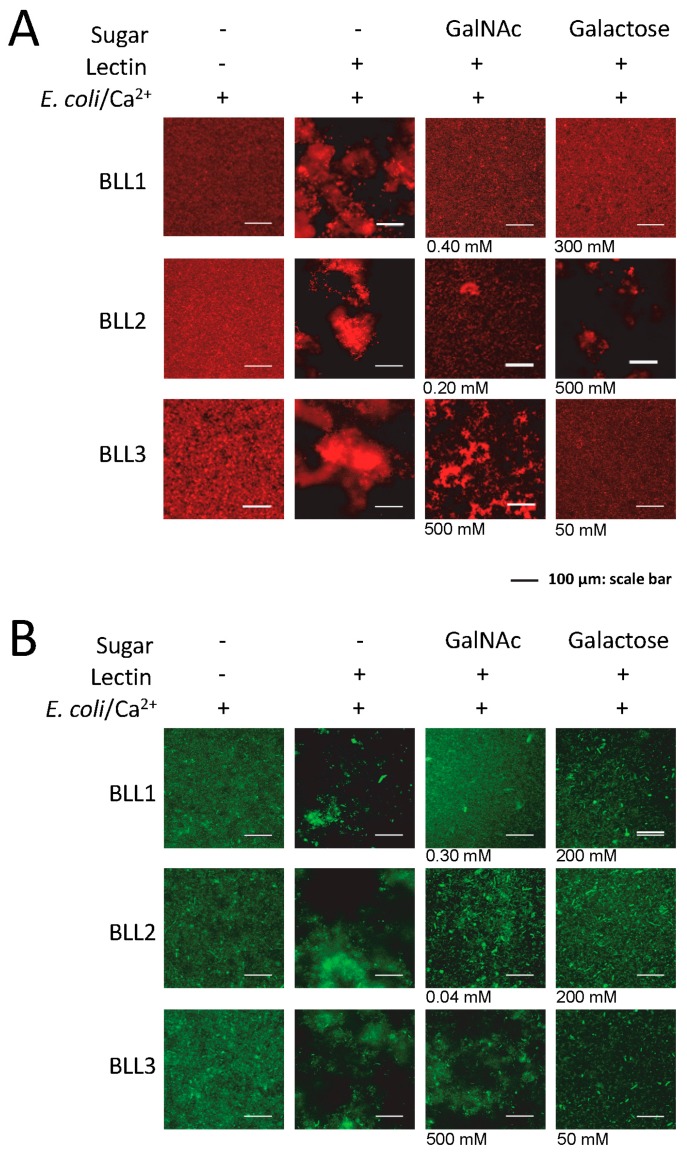
Carbohydrates’ inhibition of bacterial agglutination in the presence of the BLLs. Representative images of the agglutination of two types of bacteria ((**A**) *E. coli dt-tomato*; (**B**) *B. thuringiensis GFP*) in the presence of different concentrations of carbohydrates. In the absence of inhibition by the carbohydrate, the maximum concentrations tested are shown.

Lectin interaction is mediated by the binding of the protein to certain carbohydrates on the cell surface. We used the agglutination inhibition assay with a panel of different sugars in order to determine the carbohydrate specificity of each BLL (Figure 3 and Table 2). Under our experimental conditions, *N*-acetylgalactosamine (GalNAc) was able to inhibit the BLL1 and BLL2 agglutination at concentrations ranging from 0.04 to 0.4. In contrast, BLL3 agglutination was not inhibited with GalNAc, and competition was only observed at high concentrations of galactose.

## 4. Discussion and Conclusion

We have previously reported that the bracoviral-derived C-type lectin BLL2 from the Lepidoptera *S. exigua* contributes to its tolerance against viral infections [19]. To our surprise, the preliminary analysis showed a very high ability to induce bacterial agglutination when present, motivating a more detailed study on the agglutination properties of BLL2 and other BLL proteins.

One common property of the C-type lectins is their ability to bind to the cell surface, generally inducing agglutination of the cells. The detailed analysis of the agglutination properties of the BLLs presented here have confirmed that BLL2 is able to produce bacterial agglutination at extremely low concentrations in a Ca^2+^-dependent manner. This property was particularly evident with two different *E. coli* strains and also with *P. aeruginosa.* Although BLL2 was in general highly efficient in agglutinating Gram− bacteria, a Gram+ bacteria such as *B. cereus* was also agglutinated at very low concentration. These results are indicative that the specificity was not restricted to any of the two groups and was mediated by the surface properties other than the thickness of the peptidoglycan layer. Nevertheless, BLL2 was able to efficiently agglutinate all the tested bacteria. In general, the agglutination concentration for other C-type lectins described so far are not lower than 0.01 µg/mL [15,16,17,18,20,21], with an average value of about 1 µg/mL. To our knowledge, BLL2 is the lectin with the ability to induce bacterial agglutination at the lowest concentration described so far.

In contrast to the high activity of bacterial agglutination found for BLL2, the activity found for the other two BLLs tested here was similar to other C-type lectins. This fact is especially interesting in the case of BLL1, since the amino acid sequence was highly similar to that of BLL2. Amino acid differences between both proteins are restricted to one single residue at the CRD domain (position 100) and a few amino acids at the C-terminal end. The double-loop structure of the CRD is stabilized by disulphide bonds formed by four Cys residues conserved in all the C-type lectins [2,4]. In addition, C-type lectin oligomerization that is also mediated by disulphide bonds, is probably playing an important role in their function and selectivity [22]. It is very likely that the additional Cys residue present in BLL2 is responsible for the higher activity of this protein when compared to BLL1. Additional site mutation experiments (T100C) at BLL1 would contribute to discarding the possible role of the residues at the C-terminal end and could confirm C100 as a key determinant of the high agglutination activity in BLL2, and also in other lectins.

Early and sensitive detection of bacteria in different types of samples and materials has become of special interest in the food industry and hospital environments. Conventional methods rely on classical microbiological protocols that involve bacterial culturing and further biochemical and/or molecular characterization. Bacterial biosensors are being developed to overcome such limitations. Although the principles differ among the different methods, most of the sensors under development require a first step aimed at retaining the targeted bacterium with a ligand-coated surface [23] and a second step of identification. Lectins, such as concavalin A, have become one of the selected ligands [24]. The extremely high affinity of BLL2 against a broad spectrum of bacteria could have biotechnological applications, such as trapping bacteria in biosensors, but also in other types of applications involving bacterial capture (i.e., in air or water monitoring).

In summary, here we describe a unique C-type lectin able to interact, at extremely low concentration, with a broad range of bacteria. Additional studies are still needed to test if this property is limited to this lectin or if it could be found in paralog proteins in other Lepidoptera or bracoviruses, which could constitute an arsenal of proteins with very interesting potential industrial and biotechnological applications.

## Figures and Tables

**Table 1:** Minimum concentration (µg/mL) of BLLs producing bacterial agglutination.

Bacteria	BLL1	BLL2	BLL3
*Escherichia coli* dt tomato	0.1	1 × 10^−8^	1
*Escherichia coli*	0.1	1 × 10^−7^	1
*Pseudomonas aeruginosa*	1	1 × 10^−6^	0.1
*Salmonella entérica*	0.1	1 × 10^−3^	1
*Bacillus thuringiensis* GFP	0.1	1	200
*Bacillus cereus*	0.01	1 × 10^−4^	200
*Listeria monocytogenes*	0.1	0.1	1
*Staphylococcus aureus*	1	1	100

**Table 2:** Minimum concentration (mM) of carbohydrates inhibiting BLL-mediated bacterial agglutination.

Sugar	BLL1		BLL2		BLL3	
	Ec	Bt	Ec	Bt	Ec	Bt
D(+)-trehalose dehydrate	-	-	-	-	-	-
D(+)-maltose monohydrate	-	-	-	-	-	-
α-lactose	-	-	-	140	-	-
α-mannose	-	-	-	-	-	-
D(+)-glucose	-	-	-	-	-	-
D(+)-galactose	300	200	-	200	50	50
Sucrose	-	-	-	200	-	-
*N*-acetyl-d-galactosamine	0.4	0.3	0.20	0.04	-	-
Fructose	-	-	-	-	-	-

-: not inhibited at a concentration of 500 mM.
